# Family-based HIV prevention and intervention services for youth living in poverty-affected contexts: the CHAMP model of collaborative, evidence-informed programme development

**DOI:** 10.1186/1758-2652-13-S2-S8

**Published:** 2010-06-23

**Authors:** Arvin Bhana, Mary M McKay, Claude Mellins, Inge Petersen, Carl Bell

**Affiliations:** 1 Child, Youth, Family & Social Development Research Programme, Human Sciences Research Council, South Africa; 2 School of Psychology, University of KwaZulu-Natal, South Africa; 3 Mount Sinai School of Medicine, New York, USA; 4 HIV Center for Clinical and Behavioral Studies, New York State Psychiatric Institute and Columbia University, New York, USA; 5 Community Mental Health Council, Inc., Chicago, IL, USA, and Institute for Juvenile Research, Department of Psychiatry, School of Medicine, University of Illinois at Chicago, IL, USA

## Abstract

Family-based interventions with children who are affected by HIV and AIDS are not well established. The Collaborative HIV Prevention and Adolescent Mental Health Program (CHAMP) represents one of the few evidence-based interventions tested in low-income contexts in the US, Caribbean and South Africa. This paper provides a description of the theoretical and empirical bases of the development and implementation of CHAMP in two of these countries, the US and South Africa. In addition, with the advent of increasing numbers of children infected with HIV surviving into adolescence and young adulthood, a CHAMP+ family-based intervention, using the founding principles of CHAMP, has been developed to mitigate the risk influences associated with being HIV positive.

## Introduction

Three decades into the HIV epidemic, it is clear that HIV/AIDS is a family-based disease and that youth across the globe are particularly vulnerable. While the need for family-based HIV prevention and treatment programming is widely recognized [1], there are only a few such programmes to date that have been tested, particularly in low-resourced contexts [2]. The majority of family-based programmes internationally have focused on prevention of mother to child transmission or general child health care, educational needs or child mental health [3].

The Collaborative HIV Prevention and Adolescent Mental Health Program(CHAMP) [4] is an example of a family-focused, developmentally timed programme targeting pre- and early adolescents (9-13 years), providing a model of primary and secondary HIV prevention programme development and one that has been tested in numerous studies in the United States, sub-Saharan Africa, the Caribbean and South America.

The purpose of this paper is to provide an overview of the development and implementation of family-based programmes in poverty-affected contexts, with a particular focus on CHAMP. The aim is to draw out lessons for family-based HIV prevention and intervention programming for young adolescents, including those already infected or affected by HIV and their adult caregivers.

### Global threat of HIV

HIV infection is one of the most serious threats to the health and wellbeing of young people, and requires a continued, intensive focus on youth as they account for an estimated 45% of all new infections worldwide [5,6]. While the HIV epidemic has stabilized somewhat, the level of new HIV infections and AIDS deaths remain unacceptably high, particularly in sub-Saharan Africa [7].

The consequences of the AIDS epidemic for families can be devastating. Nearly 12 million children under the age of 18 have lost one or both parents to HIV in sub-Saharan Africa [6]. In South Africa, approximately 2.8 million children have lost at least one parent, with an estimated 1.4 million (49%) presumed to be due to AIDS [6,8]. It is estimated that 80% of children who lose a parent to AIDS are likely to have a surviving parent for whom support and care becomes critical [9]. Children orphaned by AIDS may be a particularly vulnerable group in terms of emotional problems, behavioural risk taking and school drop out [10,11].

Even in contexts where access to antiretroviral treatment (ART) and preventative interventions are more plentiful, such as the US or Europe, the HIV epidemic continues to take a toll on the health and wellbeing of children and adults. Those affected by this still life-threatening and stigmatizing disease disproportionately reside in urban communities of colour, affected by high rates of poverty, substance abuse, and exposure to community and familial violence [12,13].

In the US, for example, the majority of HIV/AIDS cases are in large inner-city communities; African Americans comprise 51% of all newly reported HIV infections, with an additional 18% accounted for by Latinos [14]. Almost one-half of the more than 40,000 new HIV infections in the US each year are among people aged 25 years and under.

Conversely, the introduction of widespread HIV counselling, testing, and ART use during pregnancy and the birth process in countries with access has led to a dramatic drop in the rate of vertical transmission [15,16]. Access to ART has also meant that many HIV-infected children who were not expected to outlive their childhood are entering adolescence [17] and are presenting with: (1) serious mental health difficulties [18,19]; (2) high-risk sexual behaviours and substance use [20-22]; and (c) non-adherence to ART [23-25]. Even brief episodes of ART non-adherence can permanently undermine treatment and lead to increased resistance to medications. Thus, perinatally infected adolescents may be living with a multidrug resistant virus and have poor health outcomes.

This grim reality becomes a serious public health issue as youth transition though adolescence, a time of increased experimentation with sexual risk behaviour and drug use. Unfortunately, few family-based programmes focused on the prevention of risk behaviour have been developed or tested with this population in high- or low-resource countries [26].

### HIV prevention and intervention efforts across the globe

Over the past three decades, there have been targeted efforts to decrease the risk for HIV infection among uninfected youth [27,28]. Despite some of the early HIV prevention efforts leading to improvements in youth knowledge regarding the significance of HIV and modes of transmission, and short-term changes in sexual risk behaviour [28-31], long-term behavioural change has been difficult to maintain [32]. Further, in a recent review of preventative interventions delivered in sub-Saharan Africa, no programme was associated with a significant decrease in actual rates of HIV infection [28,33].

As the epidemic entered its second decade, there were increasing calls for more complex models of HIV prevention and intervention programming, particularly those capable of targeting both risky and protective relational and contextual influences on youth behaviour, such as multi-level HIV prevention and care models for youth that incorporated strong partnerships with families and communities [34]. Marshalling family, social network and community-level resources around vulnerable urban youth was thought to be a critical HIV prevention and health promotion strategy [28,31,34,35].

## Case description

Although a number of family-based HIV prevention programmes have been developed and evaluated, few have actually been implemented and tested in low-resource settings where the burden of HIV exists and where the focus has been on school-based and community-based programmes targeting youth [36,3,2]. CHAMP [4,36-38] is one of the few HIV preventative efforts that was initially focused on vulnerable youth and their families in the US, and then adapted for multiple international settings.

The first family-based programme was developed in the mid-1990s based on critical streams of influence: (1) adolescent developmental models; (2) ecologically focused models that include multi-level factors (e.g., knowledge, skills and mental health characteristics of youth and their adult caregivers; interactional qualities with key protective resources, such as parents; social support systems; health-oriented institutions; and health-promoting influences of families and communities); and (3) existing empirical findings and intensive collaboration with youth, families and target community members.

### Adolescent developmental models

Initially, CHAMP embraced the developmental model with two basic views: (1) for HIV prevention to be successful, programmes need to intervene with youth prior to the initiation of sexual and drug risk-taking behaviour, specifically in pre- and early adolescence; and (2) adolescent sexual decision making occurs within social relationships and reflects a combination of social and psychological factors that need to be addressed [39].

More specifically, family and peer relationships significantly predict high-risk sexual and drug use behaviours in adolescents [40,41]. For example, family availability and monitoring are critical protective factors for reducing high-risk behaviours, while family conflict and low levels of communication are associated with increased sexual and drug use behaviour [42-44]. Also, research with youth has indicated that peers are a strong influence on sexual activity and the use of condoms, and friendships with peers who are not involved in problem behaviours are also protective factors for reduced sexual risk behaviour [12].

### Ecological theories of youth risk

As prevention efforts shifted from first generation models, a number of more complex ecological theories were employed.

The Triadic Theory of Influence (TTI) [45,46] is organized along two dimensions: levels of causation; and streams of influence. It thus represents both: (1) a theory of the problem in which the focus is on explanation and prediction of health behaviour change; and (2) a theory of action that emphasizes guiding the development of health-promoting interventions. Three relatively distinct streams of influence are proposed: intra-personal influences that contribute to: *one's self-efficacy *regarding specific behaviours; interpersonal social influences, the social situations and/or contexts that contribute to *social normative beliefs *about specific behaviours; and cultural-environmental influences, which constitute multiple socio-cultural macro-environmental factors that contribute towards *attitudes *about specific behaviours.

The theory proposes that some variables (such as intentions) have a direct effect on behaviour and are causally proximal, while others, like motivation to comply, have effects mediated through numerous other variables, such as social normative beliefs, and are considered to have a more distal influence.

The TTI has been translated into seven community field principles to provide a conceptual framework for the adaptation of CHAMP for South African uninfected youth [47-50]. The seven field principles included: (1) re-establishing the village (social networks); (2) providing access to health care (referral service); (3) improving bonding, attachment and connectedness dynamics (parenting styles and communication skills); (4) improving self-esteem (developing self-understanding and know ledge); (5) increasing social skills; (6) re-establishing the adult protective shield through monitoring (parental monitoring); and (7) minimizing residual effects of trauma (promoting supportive community networks).

Social Action Theory (SAT) [51] is an alternative model of behaviour change that also emphasizes the context in which behaviour occurs, but also refers to the developmentally driven self-regulatory and social interaction processes, and the mechanisms by which these variables result in adaptive and risky health behaviours. It was developed for uninfected populations, but has been used in studies with populations infected and affected by HIV and multiple life stressors [52,53].

Most recently, an adapted SAT model has been used to posit that HIV prevention and care outcomes for perinatally infected youth are influenced by: (1) context (e.g., family and living situation, life events, service systems); (2) self-regulation processes that promote adaptive behaviours (e.g., child capabilities and motivation factors and self-efficacy for treatment or prevention); and (3) social regulation factors (e.g., family and community support resources, caregiver supervision and involvement, social stigma of illness) [54]. This model was used to inform the development of the CHAMP+ programme within both the US and South Africa.

### Existing empirical evidence guiding youth-oriented HIV prevention

In addition to theoretical models, the CHAMP model of programme development also prioritizes basic research studies to inform interventions. More specifically, two studies - CHAMP I, a longitudinal study of 400 inner-city pre- and early adolescents living in a high sero-prevalence community, and Child and Adolescent Self-Awareness and Health (CASAH), a longitudinal study of 200 perinatally HIV-infected and 150 uninfected by perinatally HIV-exposed youth - were highly influential in informing CHAMP and CHAMP+, respectively.

CHAMP I data found that the following variables were associated with risk behaviour in uninfected youth: (1) family processes (e.g., communication, decision making, conflict, supervision/monitoring, support); (2) outside family parental support network resources; (3) youth and family HIV/AIDS knowledge and comfort discussing sensitive issue; and (4) youth communication, social problem solving, and refusal skills. Thus, the findings suggest that HIV prevention programmes targeting inner-city young adolescents need to focus on these variables in order to reduce opportunities for initiation of sexual experience and reduce risk for HIV [55].

Few HIV prevention programmes or determinant studies of behaviour exist for perinatally HIV-infected youth. CASAH was developed to identify the mental health and risk behaviour prevention needs of this population. In CASAH, high rates of psychiatric disorder were found among the predominantly African American and Latino youth living in inner-city communities, with higher rates (60%) in HIV-positive youth as compared to HIV-negative youth (47%, p = 0.05). Among the HIV-positive youth, 10% had initiated sexual behaviour, with one-third of those youth reporting unprotected sex, and among those on ART, 50% reported recent non-adherence to ART. Family variables (e.g., communication, supervision, and caregiver mental health) predicted behavioural outcomes, suggesting a need to focus family-based interventions on this population of youth to improvemental health and reduce sexual risk behaviour [19,21,22,56].

### Community collaborations

A critical component of CHAMP is the high level of intensive involvement of stakeholders in the design of the intervention for each community. Thus, within the CHAMP model of programme development, data from previous studies is placed in the hands of key stakeholders to inform the design of interventions that are culturally and contextually relevant and that can be sufficiently flexible to navigate the barriers within targeted communities. This process was used to develop the first CHAMP intervention and for subsequent iterations, including CHAMP+ [34,57,41].

Collaborative design, delivery and testing of HIV prevention programmes has been emphasized as a means of overcoming the significant obstacles to reaching vulnerable youth and their families [58]. In particular, HIV continues to be highly stigmatizing, and specific cultural concerns arise when health-related programmes are lead by "outsiders" that can significantly impede HIV prevention efforts [59]. As a result, community-based participatory research methodology has emerged as a critical research tool for developing and sustaining efficacy-based interventions.

Thus, in each context, CHAMP has consistently sought out: (1) community representatives as advice and consent givers; (2) influential community representatives as endorsers of the research programme; (3) community members as advisors (e.g., hired as front-line staff); and (4) community members as participants in the direction and focus of the research [4].

## Discussion and evaluation

### CHAMP and CHAMP+ results in the US

The CHAMP+ family-based intervention is currently delivered through multi-level group modalities, which include both multiple family sessions and parent/child group sessions. Sessions focus on: (1) parent-youth communication and decision making, particularly around sensitive topics and sexual possibility situations; (2) parental supervision and involvement; (3) family support; and (4) youth problem solving and negotiation skills. This is in addition to more traditional HIV prevention activities, including HIV knowledge.

Outcome findings available to date are summarized in multiple articles, including 17 recently published [4]. In brief, significant changes in parental reports of key family-level variables have consistently been associated with CHAMP participation relative to comparison families in the following domains: family decision making, with parents more likely to make decisions within the family for CHAMP participants; parental monitoring; family communication; and comfort related to family communication. Further, pre-adolescent youth have reported significantly less exposure to situations of sexual possibility at post-test relative to comparison youth, and parents have reported significant decreases in youth externalizing behavioural difficulties in the programme condition relative to comparison youth.

The CHAMP+ intervention represents an adaptation of the CHAMP primary prevention programme to meet the needs of HIV-positive youth and their adult caregivers. The intervention protocol focuses on: (1) the impact of HIV on the family; (2) loss and stigma associated with HIV disease; (3) HIV, health, and antiretroviral medication protocols; (4) family communication about puberty, sexuality and HIV; (5) parental supervision and monitoring related to sexual possibility situations and sexual risk-taking behaviour; (6) helping youth manage their health and medication; and (7) social support and decision making related to disclosure.

In CHAMP+, there was a clear need communicated by the target community to address issues that are specific to HIV before discussion related to family processes, such as family communication and supervision and monitoring, can proceed. Thus, HIV-specific topics, such as coping, stigma, loss, disclosure, medication taking, health and risk behaviours, were created for use with infected populations.

The adaptation process resulted in: (1) significant consumer involvement with regards to programme content; (2) strong sense of programme ownership from health care sites; and (3) high participation rates in CHAMP+. Post-intervention findings for CHAMP+ participants relative to comparison youth and adult caregivers included: increases in child reports of caregiver supervision and monitoring of peer-based activities;decreases in selected youth depression symptoms; decreases in caregiver reports of difficulties with youth; and improvements in HIV knowledge and communication about HIV with others. Manuscripts summarizing results are currently in preparation or under review and findings have been presented at multiple national and international conferences (e.g., [60]).

### CHAMP and CHAMP+ results in South Africa

South Africa adopted similar strategies to the original CHAMP and CHAMP+ in the US, namely to establish strong community and institutional partnerships so that prevention efforts are supported by communities and institutions, and to use empirical evidence reflecting relevant experiences of youth and families in the local setting to form the basis of the intervention. Key issues emerging from focused ethnographic studies for uninfected and infected South African youth [62,65] were used to inform the adaptation of the US-based programme for the South African context.

In particular, caregivers of uninfected youth in South Africa complained of disempowerment, which was a product of the erosion of traditional norms and social practices associated with protective parenting, as well as poor levels of HIV knowledge and information. A lack of trust and investment in community networks was also found to limit protective parenting in the target community [62]. For infected youth, similar psychosocial difficulties to those found in US samples emerged, with loss of biological parents to AIDS being a key issue given the late roll out of ART in South Africa [65].

In keeping with other CHAMP interventions, CHAMPSA and CHAMP+SA are developed, manualized, family group interventions focusing on intra-personal, family/interpersonal influences and community influences to strengthen family processes at each of these levels [36]. An innovation to the programmes in the South African context is the use of open-ended participatory cartoon narratives, given low literacy levels and to facilitate small group participatory experiential learning [64,66].

The CHAMPSA intervention results showed that, compared to controls, intervention families had significantly better knowledge of AIDS transmission, had less stigmatizing attitudes towards people with HIV, and talked more and had greater comfort in talking about sensitive issues to their children, as well as increased monitoring of their children. In addition, they utilised their social networks more effectively in soliciting social support [38]. Community protective influences were also strengthened through facilitating greater informal social controls and promoting social actions to create a more health-enabling community for youth [63].

Preliminary findings of the impact of CHAMP+SA suggest that families engaged with the programme reported positive experiences in helping families cope better with the diagnosis of HIV. They also reported being able to better identify problems and possible solutions [66]. Analysis of follow-up data is currently underway (Table 1).

**Table 1:** Summary of CHAMP Results

Items	Std error	Adjusted p value	Treatment group	Control group	Pooled SD Mean	Effect size mean
**CHAMPSA (Caregivers)**						
HIV transmission knowledge	0.25	0.0084	0.190	1.336	1.817	0.631
Less stigma toward HIV-infected people	0.47	0.0187	0.207	1.991	4.427	0.403
Caregiver communication comfort	0.58	0.0021	1.025	3.423	5.897	0.407
Caregiver communication frequency	0.55	0.0412	1.966	2.969	5.095	0.197
**CHAMPSA (Youth)**						
AIDS transmission knowledge	0.27	0.0647	0.88	0.12	1.54	0.50
Less stigma toward HIV-infected people	0.92	0.0045	3.96	-0.25	6.03	0.70
**CHAMP+US (Youth) Experimental control comparisons**			**F (sig)§**			
Medication support by parents			2.0*			
HIV treatment knowledge			1.9*			
**CHAMP+US (Caregivers)**						
Youth emotional difficulties			3.1*			
Youth conduct problems			2.2*			
Youth impairment			2.9*			
**CHAMP US (Caregivers)**						
Family decision making			2.1*			
Parental monitoring			5.3*			
Family communication			6.8**			
Comfort related to family communication			10.4**			
Parental perceptions of lower child behavioural difficulties			3.3*			
**CHAMP US (Youth)**						
Exposure to situations of sexual possibility			3.0*			

§Varying designs and analyses and samples in USA and South Africa preclude direct comparison of results. * p <0.05; ** p <0.01.

In each context, CHAMP is implemented by three to four facilitators who co-lead the groups, allowing for separate adult and youth sub-groups for part of the sessions. The manualized intervention allows the use of lay facilitators, such as trained parents or lay counsellors, in most settings, with or without psychologists. In South Africa, given the shortage of mental health specialists, psychologists are utilized mainly in training and supervisory capacity in keeping with the concept of task shifting suggested for low-resourced settings [67].

### Lessons learned

The development and implementation of CHAMP and CHAMP+ has suggested a number of important lessons for the field of family-based HIV prevention and mental health treatment. These include:

1. Intervention efforts are likely to be more successful and sustainable if they are collaborative in nature and involve a community advisory board that participates in the design and delivery of the intervention.

2. Universal principles of intervention based on science can be applied across continents and different contexts; yet these must be informed by local knowledge and empirical evidence to ensure cultural congruence.

3. An ecological framework within a developmental context is important in understanding complex family processes and cultural contexts, regardless of the micro-level theories used to inform specific behaviour change strategies within the ecological levels.

4. Family-based interventions should be group based to enhance social networking to enable the collective renegotiation of social norms regarding protective parenting practices.

5. Harnessing these social networks is important in fostering social support, which can enhance protective parenting, particularly in poor communities, as well as protective peer support networks for youth.

6. Social networks developed through group and community collaborative processes are important to build protective community environments, including re-building social controls to strengthen parental or adult supervision and care.

7. Lay facilitators can be successfully utilized to deliver the intervention with the support and supervision of mental health specialists in keeping with the move towards task shifting to increase access to mental health services in low-resourced settings.

## Conclusions

There is a substantive need for family-based HIV prevention and intervention programmes across the globe; yet few family-based programmes have been tested. CHAMP and CHAMP+ represent a model of family-based HIV prevention and mental health treatment that has been used across contexts (Chicago, New York, South Africa, Trinidad and Argentina) and with a range of target populations (youth in need of preventative services, HIV-positive youth, homeless youth).

Further, the resulting programmes are informed by existing empirical findings and data drawn directly from the target youth and/or families, as well as collaboration with key stakeholders. The model is based on the under-standing that in order to impact youth HIV risk outcomes (attitudes, beliefs, knowledge, behaviour), interventions need to target both risk and protective factors at the level of the child, family and context.

Using this model of intervention development, the content of the intervention can be modified to address the specific needs of youth and their families situated in unique contexts. The collaborative model of development enhances the chances that by co-designing, co-delivering and co-testing interventions with collaborative partners, including members of the target community, agency or medical setting, programmes and services can reach highly vulnerable youth and families that would otherwise be missed.

Further, the resulting efficacy-based programmes can reflect the cultural values and priorities that can be both universal and specific and ensure that programmes can be integrated into the settings they were developed for after the research phase.

## Competing interests

The authors declare that they have no competing interests.

## Authors' contributions

All authors are responsible for the gathering and interpretation of the material, the compilation of the paper, and the decision to submit the paper for publication. All authors have read the final manuscript and approved it for publication.
